# Robust Nanozyme-Enzyme Nanosheets-Based Lactate Biosensor for Diagnosing Bacterial Infection in Olive Flounder (*Paralichthys olivaceus*)

**DOI:** 10.3390/bios11110439

**Published:** 2021-11-04

**Authors:** Thenmozhi Rajarathinam, Seonghye Kim, Dinakaran Thirumalai, Sujin Lee, Minho Kwon, Hyun-jong Paik, Suhkmann Kim, Seung-Cheol Chang

**Affiliations:** 1 Department of Cogno-Mechatronics Engineering, College of Nanoscience and Nanotechnology, Pusan National University, Busan 46241, Korea; thenmozhi@pusan.ac.kr (T.R.); dinakaran@pusan.ac.kr (D.T.); 2 Department of Chemistry, Pusan National University, Busan 46241, Korea; seonghyeee@pusan.ac.kr (S.K.); isujin@pusan.ac.kr (S.L.); suhkmann@pusan.ac.kr (S.K.); 3 Department of Polymer Science and Engineering, Pusan National University, Busan 46241, Korea; mhkwon89@pusan.ac.kr (M.K.); hpaik@pusan.ac.kr (H.-j.P.)

**Keywords:** amperometric biosensor, metabolic biomarker, lactate, nanozyme–enzyme nanosheets, *Streptococcus parauberis*

## Abstract

Bacterial infections in fish farms increase mass mortality and rapid detection of infection can help prevent its widespread. Lactate is an important biomarker for early diagnosis of bacterial infections in farmed olive flounder (*Paralichthys olivaceus*). To determine the lactate levels, we designed a disposable amperometric biosensor based on Prussian blue nanozyme and lactate oxidase (LOX) entrapped in copolymer-reduced graphene oxide (P-rGO) on screen-printed carbon electrodes. Because LOX is inherently unstable, P-rGO nanosheets were utilized as a base matrix to immobilize it. After optimization in terms of enzyme loading, operating potential, and pH, the biosensor displayed maximum current responses within 5 s at the applied potential of –0.1 V vs. internal Ag/AgCl. The biosensor had Langmuir-type response in the lactate concentration range from 10 µM to 1.6 mM, a dynamic linear response range of 10–100 µM, a sensitivity of 15.9 µA mM^−1^ cm^−2^, and a lower detection limit of 3.1 µM (S/N = 3). Additionally, the biosensor featured high reproducibility, good selectivity, and stability till four weeks. Its practical applicability was tested in olive flounder infected by *Streptococcus parauberis* against the uninfected control. The results were satisfactory compared to those of a standard colorimetric assay kit, validating our method.

## 1. Introduction

Streptococcosis is a common bacterial disease affecting aquaculture farms. Streptococcosis caused by *Streptococcus parauberis* is the dominant bacterial infection in olive flounder (*Paralichthys olivaceus*), with the clinical signs of darkening of the body, exophthalmia with hemorrhage, and congestion of the spleen, kidney, liver, and heart. *S. parauberis* infection leads to devastating mortality and antibiotic resistance in the fish. Despite the use of antibacterial agents and vaccines against this pathogen, recurrent infection has been reported [1,2,3].

The detection and identification of *S. parauberis* mainly rely on the clinical symptoms [4], biochemical characteristics [2], molecular techniques including genomics [5,6,7], proteomics [8], and conventional polymerase chain reaction (PCR) based on the 23S rRNA region [9,10]. Although PCR is widely used as a standard molecular technique for the diagnosis, it is time-consuming and costly, has a high risk of contamination, and requires careful implementation to achieve sensitivity and specificity [11,12,13]. Despite the seriousness of streptococcosis in marine fishes, no efforts have been made to detect *S. parauberis* in a rapid manner. Therefore, a novel and robust technique is necessary for simple and accurate diagnosis of *S. parauberis*. Moreover, to our knowledge, there are no commercially available kits or biosensors for diagnosing bacterial infections in fish.

Metabolites are the best indicators of the current health status of an organism. Many metabolomics studies have uncovered biomarkers useful for diagnosing various infectious diseases [14]. Nuclear magnetic resonance (NMR)-based metabolomics studies have demonstrated that infection with *Aeromonas salmonicida*, a Gram-negative bacterium, induces metabolic changes in lipoproteins and choline-based residues [15]. Several studies have investigated metabolic changes in tilapias upon infection by *Streptococcus* species, and revealed that *N*-acetylglucosamine, leucine, and proline could be biomarkers for streptococcosis [16,17,18].

According to NMR metabolic profiling, lactate level is elevated (hyperlactatemia) in *S. parauberis*-infected olive flounder, and therefore the detection of lactate biomarker contributes to streptococcosis infection diagnosis at an early stage. Previously reported conventional analytical methods for detecting lactate in fishes include liquid chromatography-tandem mass spectrometry [19] and ^1^H NMR spectroscopy [20]. Although these methods are reliable, they have significant demerits such as the need for expensive equipment or expertise, multi-step sample pre-preparation, high time consumption, the inability for real-time monitoring, and increased risk of false-positive responses due to potential interferents in biological matrices. In comparison, electrochemical biosensing techniques are rapid, and free from these drawbacks [21]. Thus, they have the potential for accurate real-time quantification of lactate. Enzymatic biosensors are highly desirable for the selective detection of lactate amongst various interfering compounds. A flavoprotein enzyme, lactate oxidase (LOX), is generally employed as the catalyst to fabricate amperometric lactate biosensors. As shown in Equation (1), LOX catalyzes the conversion of lactate to pyruvate, and the formed hydrogen peroxide (H_2_O_2_) can be easily detected by the sensing electrode [22].

(1) $$Lactate+O_{2}+H_{2}O\;\overset{LOX}{\to }\;Pyruvate+H_{2}O_{2}$$

(2) $$H_{2}O_{2}\to O_{2}+2H^{+}+2e^{-}$$

The byproduct H_2_O_2_ provides an indirect estimation of the lactate concentration. Although much research has been conducted on H_2_O_2_ oxidation (Equation (2)) at large overpotentials (around +0.4 V/+0.6 V), we would like to polarize the working electrode in a less negative potential region (−0.1 V) to study the reduction kinetics. To lower the working potential, artificial peroxidases and/or redox mediators can be used for signal amplification. Specifically, Prussian blue (PB), could be incorporated as a peroxidase mimic for electrochemical biosensors due to their excellent catalytic activity and facile synthesis the integration of PB nanozyme helps the sensing of H_2_O_2_ at lower applied potentials [23]. More importantly, redox mediators such as PB can improve lactate detection at micromolar concentrations with higher sensitivity and at a mild applied potential [24,25]. Notably, PB demonstrates enhanced stability with oxidase enzymes having optimum alkaline pH (pH 7.4), and it could significantly lower the interference from ascorbic acid [26].

Despite many studies of enzymatic lactate biosensors, there is still considerable interest in improving the stability properties of biosensors. Since LOX is a less stable enzyme, further stabilization of the enzyme layer is mandatory. To immobilize LOX structures onto the electrode interface, many researchers have employed nanomaterials such as nitrogen-doped carbon nanotubes [27], mesoporous silica [28], ceria nanoparticles [29], and copper metallic frameworks [30]. Polymer binders such as chitosan and Nafion analogs could be utilized as an additional layer to prevent LOX inactivation and efflux. Although these dense polymers seem to be connected to higher noise in chronoamperometry (CA) experiments and probably cause lag responses, at the optimum concentrations they not only act as a barrier against potential interferents but also provide a suitable framework for enzyme orientation [30]. Therefore, the sensing interface should be configured with a nanomaterial and a polymer binder for better enzyme orientation and improved stability.

**Scheme 1:**
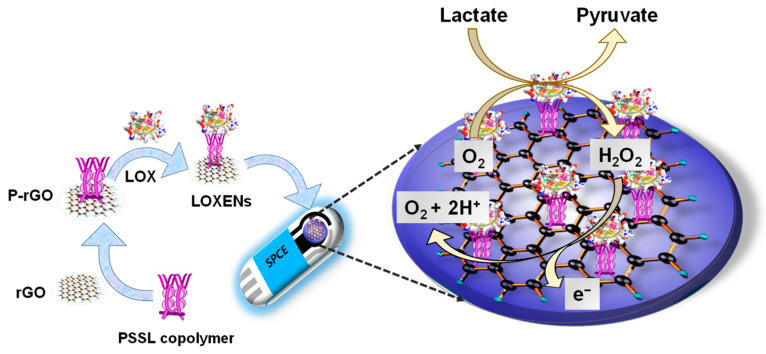
Design and mechanism of the proposed lactate biosensor.

In this study, we used NMR-based metabolomics analysis to identify metabolite biomarkers in the spleen of olive flounder infected with *S. parauberis*. Significant changes in metabolites were observed, particularly lactate, whose elevation enables the diagnosis of *S. parauberis* infections. Next, to rapidly evaluate the lactate concentration, we designed an amperometric lactate biosensor based on screen-printed carbon electrodes (SPCEs). First, the SPCEs were modified with PB nanozyme (PB/SPCEs). Meanwhile, reduced graphene oxide (rGO) was dispersed in a synthesized copolymer binder, poly(sodium 4-styrenesulfonate-r-LAHEMA) PSSL), and the product was termed P-rGO nanosheets. The P-rGO nanosheets were mixed with LOX to create affinity or electrostatic interactions between the two components. In the resulting mixture (termed LOXENs), the P-rGO nanosheets not only provide a large surface for entrapping abundant LOX but also prevent enzyme efflux, thus enhancing the stability. The LOXENs was simply cast over the nanozyme-modified biosensor. A simple representation of the designed lactate biosensor, referred to as LOXENs/PB/SPCE, is shown in Scheme 1. Overall, the nanozyme–enzyme nanosheet modification is expected to improve the analytical performance of the amperometric lactate biosensor.

## 2. Experimental

### 2.1. Reagents and Chemicals

The following reagents and chemicals were procured from Sigma-Aldrich (USA): lactate oxidase (LOX, EC 1.1.3.2) from *Aerococcus viridans* species, sodium l-lactate, potassium ferricyanide (K_3_[Fe(CN)_6_]), 4-dimethylaminopyridine (DMAP), iron (III) chloride (FeCl_3_), sodium 4-styrenesulfonate (SS), 2-hydroxyethyl methacrylate (HEMA), sodium phosphate monobasic (NaH_2_PO_4_), hydrazine monohydrate, sodium phosphate dibasic (Na_2_HPO_4_), sodium chloride (NaCl), azobisisobutyronitrile (AIBN), N,N′-dicyclohexylcarbodiimide (DCC), lipoic acid (LA), dichloromethane (DCM), dimethyl sulfoxide (DMSO), magnesium sulfate (MgSO_4_), and potassium chloride (KCl). Graphene oxide (GO) was obtained from Nanosolution (Jeonju, Jeonbuk, South Korea). Phosphate buffer solution (PBS) at a concentration of 50 mM and various pH was prepared using NaH_2_PO_4_, Na_2_HPO_4_, and KCl [29]. All other reagents employed were of standard or analytical grade and used without any further pretreatment steps. The aqueous solutions were prepared using triple distilled water (18 MΩ) from a Milli-Q water purification system.

### 2.2. Construction of Lactate Biosensors

To prepare LOXENs, equal volumes of P-rGO (0.2 mg mL^−1^, for the synthesis see Section S2 in Supplementary material) and LOX (25 IU mL^−1^) were mixed and left for 10 min. The surface area of P-rGO was large enough to entrap a significant amount of LOX. Approximately 6 µL of the prepared LOXENs was drop-casted onto the SPCE surface. Prior to drop-casting, the SPCE was modified with the nanozyme (Section S1 in Supplementary material) following our previous report [31]. The obtained biosensors, LOXENs/PB/SPCEs, were finally dried at 4 °C for 12 h before use. The PSSL copolymer contained in P-rGO has adhesive/binding properties that promote LOX loading by simple adsorption.

### 2.3. Equipment and Measurements

Morphological analysis of the biosensors was conducted using field-emission scanning electron microscopy (FE-SEM, Zeiss SUPRA 25). CA evaluations were carried out using electrochemical equipment (Compactstat, Ivium Technologies B.V., Eindhoven, The Netherlands).

For CA, the biosensor was attached to a potentiostat, 60 μL of PBS was placed onto the sensing surface, and then a potential of −0.10 V was applied. After achieving a stable baseline response, 20 μL of lactate solution was introduced, and the resulting current responses were measured over time. To acquire the calibration curve, the evaluations were repeated using standard lactate solutions at various concentrations.

### 2.4. Experimental Fish and S. parauberis Challenge

Olive flounder with an average body weight of 40 g was obtained from a commercial fish farm in South Korea and acclimatized to laboratory conditions for 2 weeks in a 1000 L tank with circulated seawater at 26 °C. *S. parauberis* strain SpOF-3k serotype I was cultured for 24 h on brain heart infusion agar (BHIA) containing 1% NaCl. For *S. parauberis* infection, the fish (*S. parauberis* infection group, n = 50) was subcutaneously injected with bacteria at a final concentration of 4.3 × 10^3^ colony-forming units/fish. The control group (n = 50) was injected with the same volume of sterile saline. Water temperature was maintained at 26 °C during the experiment. At 7 days post-challenge (DPC), the cumulative mortality was 38% and 0% in the *S. parauberis*-infected and control groups, respectively. Twenty fish per group were dissected at 7 DPC after controlled anesthesia with MS-222. The target organ (spleen) from both groups was removed aseptically and frozen immediately using liquid nitrogen.

The presence of infection was confirmed by homogenizing the isolated spleen followed by serial dilution and culturing on BHIA for 24 h. No infection was found in the control group, and fish from the infected group were confirmed to be severely infected with *S. parauberis*.

### 2.5. ^1^H NMR Analysis

The spleen samples were lyophilized and homogenized individually for metabolite extraction. Within the *S. parauberis*-infected group and the control group, the homogenized samples were pooled (9 and 10 samples, respectively) to achieve a weight of 60 mg on average. Metabolite extraction was performed on each pooled sample using Bligh and Dyer’s method [32]. The polar phase supernatant of each sample was collected and lyophilized. Later, the samples were re-dissolved in 2 mM 3-(trimethylsilyl) propionic-2,2,3,3-d_4_ acid sodium salt (TSP-d_4_) solution in 700 μL of D_2_O, and finally transferred into a 5 mm NMR tube. All samples were analyzed using a 600 MHz Agilent NMR spectrometer (Agilent, Santa Clara, CA, USA). A Carr–Purcell–Meiboom–Gill pulse (CPMG) sequence was used to suppress the peaks of water and macromolecules. Spectra were acquired with a 22.375 μs 90° pulse, 1 s relaxation delay, 3 s acquisition time, and 128 scans. The total acquisition time was 9 min 56 s.

### 2.6. Preparation of Fish Spleen Extracts for Biosensor and Kit Tests

The practical utility of the fabricated biosensor was examined following a standard addition method employing spleen extracts from both the control and *S. parauberis*-infected groups. The samples were prepared at various dilutions with 50 mM PBS (pH 7.4). Diluted samples with minimal background current and noise were chosen to minimize matrix effects and possible interferences. Spleen extracts (4 × 60 μL) spiked with 60 μL of lactate at known concentrations (0.0, 50, 100, and 150 μM) were prepared and subjected to CA measurements. The dilution factor of the spiked samples was 0.5. The cumulative lactate concentrations detected in spiked samples were calculated in the same way as for the linear calibration plots.

To compare to the biosensor results, the lactate concentrations in control and *S. parauberis*-infected groups were also estimated using a standard colorimetric lactate assay kit (cat. ab65331; Abcam) according to the manufacturer’s protocol.

### 2.7. Data Analysis

Identification and concentration estimation of the metabolites were performed using Chenomx NMR suite 8.4 (Chenomx Inc., Edmonton, AB, Canada). The concentration of metabolites was calculated with reference to the 2 mM TSP peak. MetaboAnalyst 5.0, a web-based metabolomics analysis platform, was used for *t-*test analysis adjusted to a false discovery rate (FDR) and receiver operating characteristic (ROC) analysis, to assess the diagnostic performance of the metabolite.

## 3. Results and Discussion

### 3.1. Lactate Levels in S. parauberis-Infected Fish Spleen

**Figure 1:**
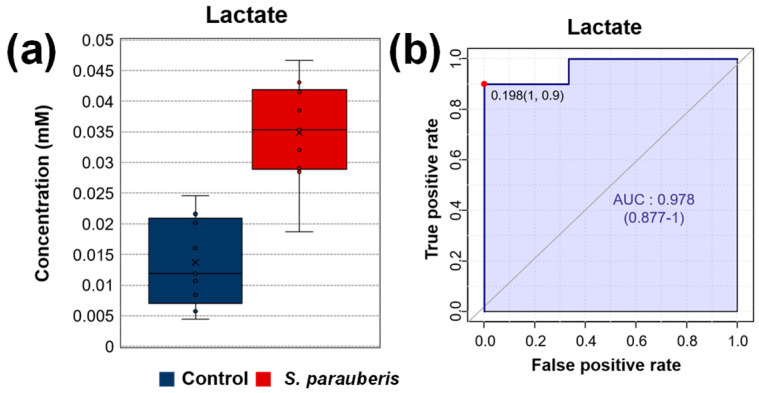
(**a**) Concentrations of lactate as potential biomarker identified through NMR spectroscopy. (**b**) ROC analysis result for lactate.

A total of 21 metabolites were identified and quantified in the spleen of olive flounder as given in Table S1. These metabolites were further analyzed using the *t*-test, and significant increases in lactate were observed in the *S. parauberis*-infected group compared to the control (FDR < 0.05). Figure S1 shows the lactate NMR peaks characteristic of control and *S. parauberis* infected groups. Lactate is represented as doublet peak at 1.33 ppm (Figure S1a and quartet peak at 4.1 ppm (Figure S1b) in the ^1^H-NMR spectra. The average lactate concentration in the control group was below 0.002 mM, whereas that in the *S. parauberis*-infected group increased by at least 2-fold as shown in Figure 1a. The area under the curve (AUC) values from the ROC were used to determine the diagnostic performance of lactate. The results showed excellent ability to predict lactate with a high AUC value of 0.978 (Figure 1b). The results are summarized in Table 1. The *p* value of ROC was also significant (*p* = 1.08 × 10^−4^). Therefore, we confirmed that lactate is an effective biomarker for detecting *S. parauberis* infection, and its concentration could distinguish the infected fish from control.

### 3.2. Physicochemical Characterization

**Figure 2:**
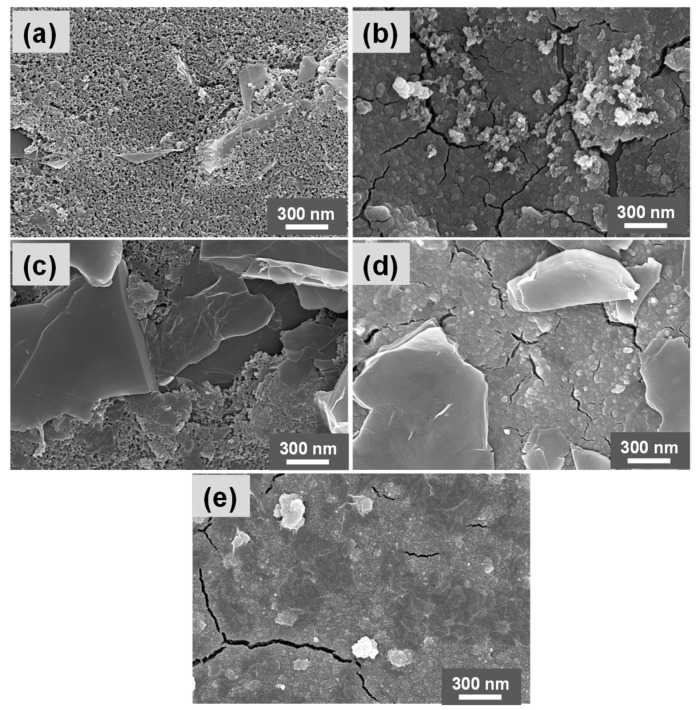
FE-SEM images of (**a**) bare SPCE, (**b**) PB/SPCE, (**c**) P-rGO/SPCE, (**d**) P-rGO/PB/SPCE, and (**e**) LOXENs/PB/SPCE.

The morphology of biosensors after successive modifications was characterized by FE-SEM (Figure 2). The bare SPCE displayed a relatively rugged surface and an uneven morphology, whereas the surface of nanozyme-modified SPCE was slightly smoother with uniformly distributed PB nanoparticles (Figure 2a,b). On P-rGO/SPCE, the two-dimensional rGO nanosheets were distributed evenly, except for some slight aggregates (Figure 2c). Both PB nanoparticles and rGO nanosheet-like structures were observed on P-rGO/PB/SPCE (Figure 2d). After LOXENs immobilization, the surface became predominantly smooth with small globe-like structures (Figure 2e). This globular morphology suggests good interactions between the P-rGO nanosheets and LOX.

### 3.3. Electrochemical Characteristics of Biosensors

**Figure 3:**
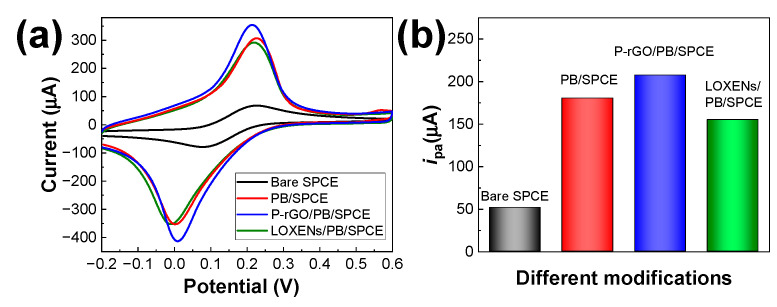
(**a**) CV results of the modified biosensors (bare SPCE, PB/SPCE, P-rGO/PB/SPCE, and LOXENs/PB/SPCE) in 5 mM K_3_[Fe(CN)_6_] prepared in 0.1 M KCl at 50 mV s^−1^. (**b**) The corresponding *i*_pa_ values plot.

The modified biosensors (bare SPCE, PB/SPCE, P-rGO/PB/SPCE, and LOXENs/PB/SPCE) were electrochemically characterized by CV experiments. The Figure 3a represents the cyclic voltammograms of the biosensors in 5 mM K_3_[Fe(CN)_6_] prepared in 0.1 M KCl at the sweep rate of 50 mV s^−1^. In addition, the anodic peak currents (*i*_pa_) were measured and plotted for the modified biosensors (Figure 3b). The *i*_pa_ values for bare SPCE, PB/SPCE, P-rGO/PB/SPCE, and LOXENs/PB/SPCE were 51.7, 180.4, 207.6, and 155.7 μA, respectively. All the modified biosensors including bare SPCE revealed distinguished redox peaks. The PB/SPCE, P-rGO/PB/SPCE, and LOXENs/PB/SPCE showed increased *i*_pa_ values, indicating that the stepwise modifications of PB, P-rGO, and LOXENs improve the specific surface area of the biosensor as compared with bare SPCE. In comparison with P-rGO/PB/SPCE, the LOXENs/PB/SPCE exhibited a slightly decreased *i*_pa_ value due to the presence of a relatively large enzyme molecule, that partially impedes electron transfer between the electrolyte and the biosensor. In addition, it confirms the successful formation of the LOXENs on the nanozyme modified biosensors.

**Figure 4:**
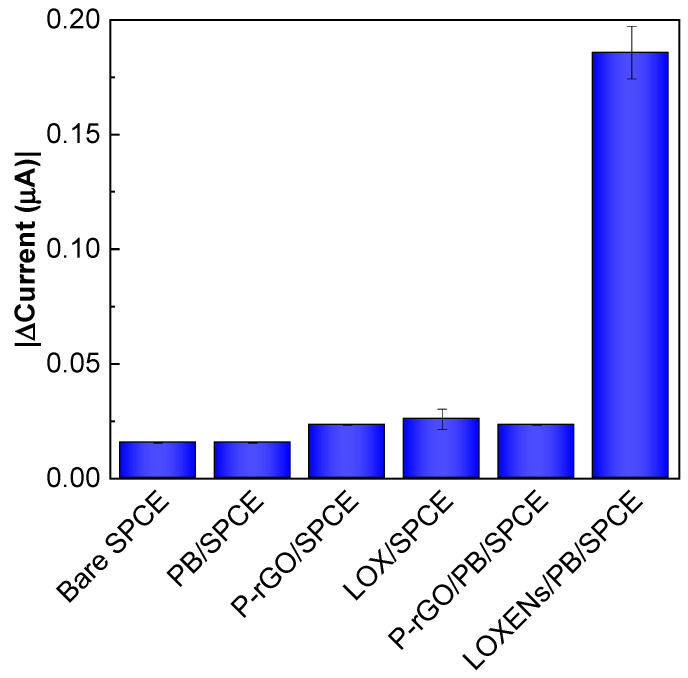
CA current changes of biosensors in 50 mM PBS (pH 7.4) after adding 100 µM lactate.

Various biosensors (bare SPCE, PB/SPCE, P-rGO/SPCE, LOX/SPCE, P-rGO/PB/SPCE, and LOXENs/PB/SPCE) were electrochemically characterized by CA experiments using 100 µM lactate at an operating potential of −0.10 V. The CA current responses were measured 10 s after lactate addition, and the results are shown in Figure 4. Only LOXENs/PB/SPCE showed a significant increase in the current responses upon lactate addition. Even the biosensor modified with LOX alone (LOX/SPCE) had current responses similar to those of bare SPCE. In contrast, LOXENs/PB/SPCE demonstrated maximum current responses due to the presence of P-rGO, which provided biocompatibility and retained the bioactivity of LOX.

### 3.4. Optimization of Biosensor Performance

**Figure 5:**
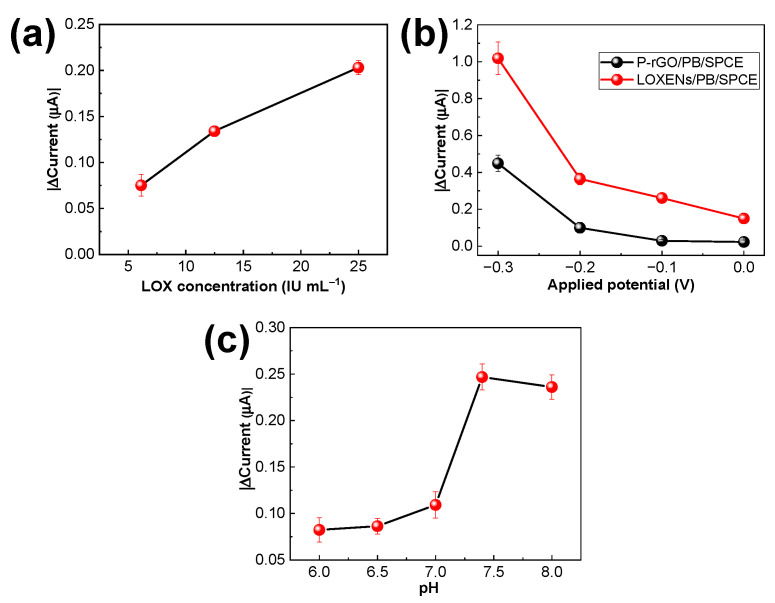
(**a**) Current changes in response to 100 µM lactate for LOXENs/PB/SPCE fabricated using various loadings of LOX. (**b**) Current changes under various applied potentials for P-rGO/PB/SPCE and LOXENs/PB/SPCE in 50 mM PBS (pH 7.4). (**c**) Current changes to 100 µM lactate solution prepared in PBS (50 mM, various pH) for LOXENs/PB/SPCE.

The LOXENs/PB/SPCE biosensors were further optimized in terms of the LOX enzyme loading, operating potential, and solution pH. Enzymes as macromolecules may impede electron transfer and compromise the sensitivity of biosensors. To optimize the LOX loading, biosensors were prepared by using various concentrations of LOX (6.125, 12.5, and 25.0 IU mL^−1^) with the P-rGO nanosheets. From the CA measurements (Figure 5a), the amperometric current responses increased as the enzyme concentration increased from 6.125 to 25.0 IU mL^−1^. Thus, 25.0 IU mL^−1^ was selected because of its increased bioactivity.

Next, the amperometric experiments were repeated using 100 µM lactate on P-rGO/PB/SPCE and LOXENs/PB/SPCE in the applied potential range of 0.0 to −0.30 V. As shown in Figure 5b, P-rGO/PB/SPCE exhibited only slight changes in its cathodic current response to lactate at −0.2 and −0.3 V. For LOXENs/PB/SPCE, the cathodic current response drastically increased as the applied potential became more negative. However, to minimize the influence of possible interferents at negative overpotential, a very low operating potential of −0.1 V was chosen.

Most enzymes have an optimal pH value at which they form suitable enzyme-substrate complexes and display maximal catalytic activity. In addition, it is well recognized that pH affects the enzyme conformation, which can diminish the enzyme’s affinity for the substrate. Therefore, the effect of pH on LOXENs/PB/SPCE was tested using 50 mM PBS at pH = 6.0–8.0. Upon the addition of lactate, maximum current responses were only observed near the neural pH (pH 7.4) instead of other pH values (Figure 5c). Moreover, arginine residues in the enzyme likely have a positive charge at near neutral pH, which would promote the enzyme’s affinity with the negatively charged PSSL copolymer backbone in P-rGO. In consideration of the enzyme stability and maximum bioactivity at physiological pH, pH 7.4 was chosen as the most suitable condition. Another study also reported that LOX shows maximum catalytic performance and stability at pH 7.4 [33], in accordance with our results.

### 3.5. Calibration Plots

**Figure 6:**
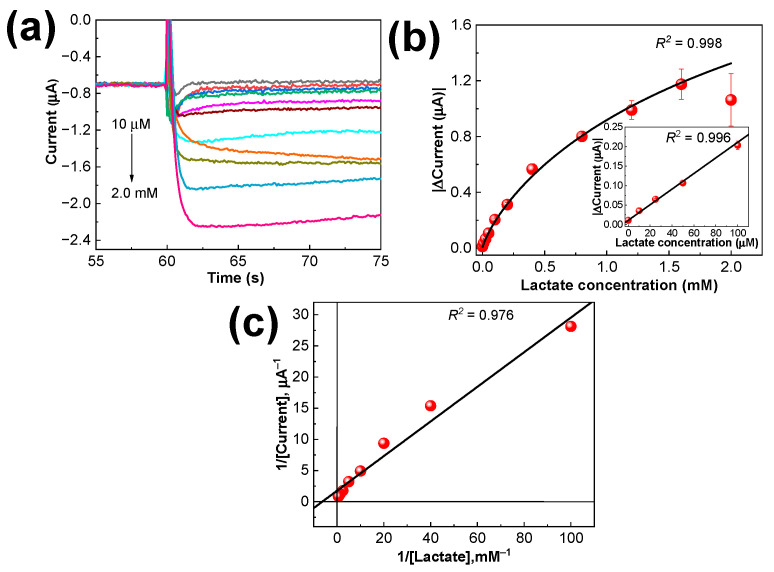
(**a**) Amperometric results of LOXENs/PB/SPCEs at −0.1 V in 50 mM PBS (pH 7.4) with successive additions of lactate. (**b**) Fitting the Langmuir model to the current responses (measured 10 s after lactate addition) to a maximum of 1.6 mM lactate. Inset: Calibration curve for lactate concentrations up to 100 µM. (**c**) Lineweaver–Burk model fitting to the catalytic region using the current responses to lactate additions up to 1.6 mM (n = 4).

CA measurements were carried out for various concentrations of lactate (0.0, 0.01, 0.025, 0.05, 0.1, 0.2, 0.4, 0.8, 1.2, 1.6, and 2.0 mM) using LOXENs/PB/SPCE under the optimized conditions. As shown in Figure 6a, the current responses measured 10 s after lactate addition were stable and proportional to the added lactate concentration.

At higher lactate concentrations, however, the amperometric current responses became saturated. This saturation phenomenon due to lactate adsorption onto the nanozyme–enzyme nanosheet surface could be described by the Langmuir isotherm model. When the non-linear current responses were fitted with Langmuir isotherm model as given in Figure 6b and a high *R^2^* value of 0.998 was obtained over a broad dynamic range of lactate concentration from 10 μM to 1.6 mM. LOXENs exhibits two discrete regions with regard to lactate concentration: namely the catalytic and inhibition regions. The initial catalytic region (10 μM to 1.6 mM lactate) results from a first-order reaction, and the reaction rate in this region is linear to the substrate concentration. A decreased current response was observed at 2 mM, which corresponds to a zero-order reaction. Therefore, the second region demonstrates an inhibition effect linked to the substrate, similar to a work reported elsewhere [34].

A linear calibration curve was constructed using data from the initial catalytic region (inset of Figure 6b). The plotted results are the average values of four measurements, with error bars indicating their standard deviation. The linear dynamic range was estimated to be 10–100 µM, and the regression equation is *i* (µA) = 0.002*C_lactate_* (µM)–0.0695, with *R*^2^ = 0.996 and a sensitivity of 15.9 µA mM^−1^ cm^−2^. The limit of detection (LOD) was 3.1 µM (S/N = 3). We calculated the limit of detection using the formula 3σ/*m,* where σ is the standard deviation of the blank and *m* is the slope obtained from the calibration curve using a standard approach [35]. Table 2 compares the analytical parameters of other amperometric lactate biosensors with our results. The sensitivity and LOD of our biosensor were comparable to the studies reported in Table 2. Additionally, the biosensor response (<10 s) was much faster than that in previous reports.

### 3.6. Kinetic Study of LOXENs

For lactate concentrations (10 µM to 1.6 mM), the biosensor response for the catalytic region was examined with the Michaelis–Menten kinetics. The Michaelis–Menten constant is an important parameter for determining the enzyme-substrate reaction kinetics, and it can be calculated using the Lineweaver–Burk equation as follows [32]:

(3) $$\frac{1}{i}=\frac{1}{i_{max}}+\frac{K_{M}}{i_{max}}\frac{1}{\left[S\right]}$$

where *i* is the current, *i*_max_ is the maximal current, *K_M_* is the Michaelis–Menten constant, and *S* is the substrate concentration. From the plot of 1/*i* vs. 1/[*S*] (Figure 6c), the two parameters were evaluated from the slope and intercept to be *i*_max_ = 4.53 µA and *K_M_* = 0.158 mM. The obtained *K_M_* was lower than the previously reported value of 1.05 mM [34]. This decreased *K_M_* indicates retention of the original LOX conformation in LOXENs/PB/SPCE, which contributes to the higher affinity and activity of LOXENs toward the reaction of lactate.

### 3.7. Interference Study

**Figure 7:**
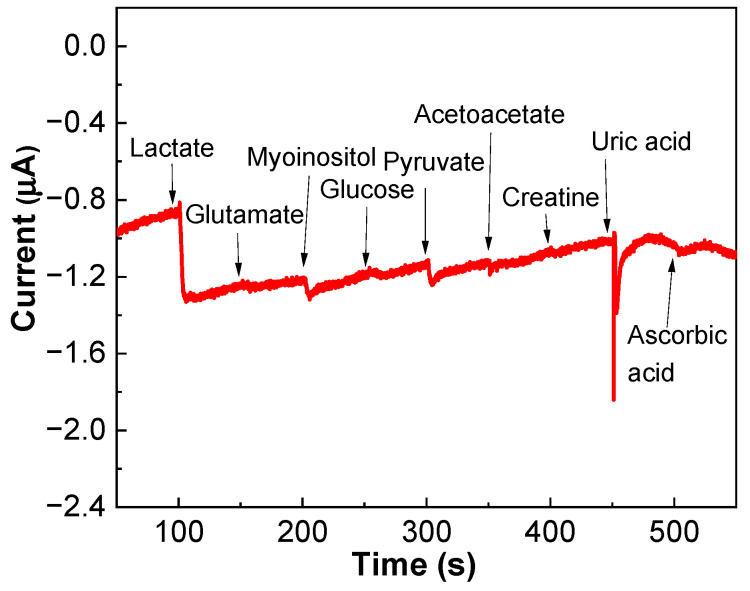
Chronoamperometric results of LOXENs/PB/SPCE in PBS (pH 7.4) at –0.1 V, following successive addition of 100 µM each of lactate, glutamate, myoinositol, glucose, pyruvate, acetoacetate, creatine, uric acid, and ascorbic acid.

A biosensor’s resistance against interference is important for practical applications. Therefore, we tested the lactate sensing ability of LOXENs/PB/SPCE in the presence of common interfering species (100 µM each) at an applied potential of −0.1 V. According to Figure 7, the biosensor displayed rapid current response to lactate that was not impacted by co-existing interferents such as glutamate, glucose, acetoacetate, and creatine due to the substrate specificity. The additions of myoinositol, pyruvate, uric acid, and ascorbic acid exhibited less than 10% interference and therefore did not affect lactate detection. Although, on uric acid addition, a moderate level of noise and non-faradaic responses were recorded, it did not affect lactate detection. Moreover, it was reported that the mild operating potential of −0.1 V efficiently suppresses the interferences due to uric acid and ascorbic acid [44]. In particular, the anti-interference property of the biosensor is attributed to the electronegative sulfonate and disulfide functionalities in the PSSL copolymer backbone by actively repelling negatively charged species like uric acid and acetoacetate. These results suggest the possibility of selective lactate detection using LOXENs/PB/SPCE.

### 3.8. Reproducibility and Stability

Four individual LOXENs/PB/SPCE biosensors were employed to evaluate the reproducibility. The relative standard deviation was found to be 2.05% from four repeated measurements of the four biosensors, as shown in Figure S2a. The stability of the biosensors was assessed via CA experiments using 100 µM lactate. The biosensors were stored at 4 °C for up to 4 weeks and tested weekly (n = 2). The loss of initial current response was 8.6%, 15.9%, 19.2%, and 38.1% after 1, 2, 3, and 4 weeks. Even after 4 weeks, the sensor retained nearly 62% of the current response, proving the stability of nanozyme and LOXENs components in the biosensor (Figure S2b). Therefore, the biosensor exhibited good reproducibility and stability toward lactate detection.

### 3.9. Real Sample Testing

The practical applicability of the proposed biosensor was evaluated by determining the lactate concentration in spleen extracts from both the control and *S. parauberis*-infected groups of olive flounder using the standard addition method. The CA method for constructing the lactate calibration curve was followed, except that the spleen extracts replace the standard lactate solution. The results are presented in Table 3. The measured lactate concentration in the control group was 8.40 µM, whereas that in the *S. parauberis*-infected group was 29.9 µM (n = 4). The recovery percentage for the spiked infected samples was nearly acceptable and yet more studies are required. The lactate level in infected fish was approximately 3.5 times that of the control, suggesting increased lactate concentrations in the *S. parauberis*-infected group.

The reliability of the biosensor was verified by comparing the results to a standard calorimetric lactate assay kit. Following the standard kit protocol, a standard curve was obtained (Figure S3), which was used to determine the lactate concentrations of the control and *S. parauberis*-infected groups to be 10.2 and 27.2 µM, respectively. These values agree well with results measured using the biosensor, proving the feasibility of the biosensor for lactate detection in practical applications.

## 4. Conclusions

A simple and rapid lactate biosensor was fabricated based on nanozyme–enzyme nanosheets and successfully used to determine the lactate levels in *S. parauberis*-infected olive flounder. The conformation of LOX on the prepared LOXENs/PB/SPCE resulted in enhanced stability and biocompatibility. The biosensor features an improved sensitivity (15.9 μA mM^−1^ cm^−2^), a linear range of 10–100 μM, and a lower LOD (3.1 µM) for lactate detection. The mild operating potential used for detection (−0.1 V) not only enhances the selectivity toward lactate but also reduces the impact of easily oxidizable interferents. Additionally, the biosensor exhibited Langmuir-type responses up to a lactate concentration of 1.6 mM. The developed biosensor remained very stable over four weeks of storage and had high reproducibility. Moreover, the measurement results agreed with those obtained using the standard colorimetric lactate assay kit, proving the reliability of the biosensor. This fabricated biosensor is therefore an ideal tool for the rapid diagnosis of *S. parauberis* infection at fish farms.

## Figures and Tables

**Table 1:** Comparison of lactate concentration in the spleen of control and *S. parauberis*-infected olive flounder.

Metabolite	Concentration (mM, Mean ± SD)		*p* Value Adjusted by FDR	ROC Analysis		
	Control	*S. parauberis*		AUC (95% CI)	Sensitivity	Specificity
Lactate	0.014 ± 0.006	0.035 ± 0.008	1.59 × 10^−4^	0.978 (0.877−1)	90%	100%

SD: standard deviation, FDR: false discovery rate, ROC: receiver operating characteristics, AUC: area under the curve.

**Table 2:** Comparison of the state-of-the-art analytical performance of various amperometric lactate biosensors.

Biosensors	Operating Potential vs. Ag/AgCl (V)	Linear Range (μM)	LOD (µM)	Response Time (s)	Sensitivity (µA mM^−1^ cm^−2^)	References
LOX/PtNp–CNF–PDDA/SPCEs	0.50	25–1500	-	11	36.8	[36]
LOX–FSM8.0/Naf/CoPC–SPCEs	0.45	18.3–1500	18.3	90	4.54	[28]
LOX/Pt/SPCE	0.60	0–1000	0.5	50	0.448	[37]
HRP/LOX/SPCE	–0.10	10–180		-	0.00084	[38]
LOX/albumin–mucin hydrogel/Pt disc	0.65	0.70–1500	0.7	90	0.79	[22]
LOX/poly (An-co-FAn) film	0.40	100–5500	100	50	1.18	[39]
LOX/Polyelectrolyte/Porous carbon electrode	0.60	75–1000	10	20–60	15.59	[40]
PB/LOx/chitosan coated gold fiber	−0.10	0–30,000	137	-	19.13	[41]
LOx/TTF/MWCNT/Printed tattoo electrode	0.050	0–20,000	-	continuous 30 min	10.31	[42]
LOX–chit–CNT/TTF–CNT/Au	−0.17	0–24000	-	-	68	[43]
LOXENs/PB/SPCE	−0.10	10–100	3.1	10	15.9	This work

PtNp: platinum nanoparticle, CNF: carbon nanofibers, PDDA: poly(diallyldimethylammonium) chloride, FSM8.0: mesoporous silica, Naf: Nafion, CoPC: cobalt phthalocyanine, HRP: horseradish peroxidase, poly (An-co-FAn): poly(aniline-co-fluoroaniline), TTF: tetrathiafulvalene, MWCNT: multiwalled carbon nanotubes, chit: chitosan, CNT: carbon nanotubes, Au: gold.

**Table 3:** Comparison of lactate concentrations measured in control and *S. parauberis*-infected fish spleen samples using the biosensor and the colorimetric assay kit.

Methods	Samples	Added (µM)	Found ± SD (µM)	Recovery (%)	RSD (%)
Proposed biosensor	Control group	0	8.40 ± 2.4	-	28.6
		50	116.3 ± 6.0	199.2	5.1
		100	181.6 ± 5.2	167.6	2.8
		150	229.4 ± 7.2	144.9	3.1
	*S. parauberis* infected group	0	29.9 ± 8.2	-	27.4
		50	129.3 ± 2.7	161.8	2.1
		100	237.4 ± 2.7	182.7	1.1
		150	303.0 ± 2.7	168.4	0.9
Colorimetric assay kit	Control		10.2		
	*S. parauberis* infected group		27.2		

SD: standard deviation, RSD: relative standard deviation.

## Data Availability

The data presented in this study are available on request from the corresponding author.

## Supplementary Materials

The following are available online at https://www.mdpi.com/article/10.3390/bios11110439/s1, Section S1: PB electrodeposition. Section S2: Synthesis of P-rGO. Figure S1: ^1^H-NMR spectra of spleen samples from the control (blue line) and *S. parauberis* infected fish (red line). (**a**) Lactate is represented as doublet peak at 1.33 ppm (1.326 and 1.338 ppm) and (**b**) quartet peak at 4.1 ppm in the ^1^H-NMR spectra, Figure S2: (**a**) Reproducibility of LOXENs/PB/SPCE in 100 μM lactate prepared in 50 mM PBS (pH 7.4). The error bars indicate the mean of four replicate measurements using each of four individual biosensors. (**b**) Stability of LOXENs/PB/SPCE after storage at 4 °C over a period of 4 weeks, Figure S3: Typical calibration curve of lactate standard obtained using calorimetric assay kit after background signal subtraction (n = 2 ± SD), Table S1: The concentrations of metabolites acquired by ^1^H-NMR spectroscopy from the spleen of olive flounder.
