# Supplementary material for: Robust Nanozyme-Enzyme Nanosheets-Based Lactate Biosensor for Diagnosing Bacterial Infection in Olive Flounder (Paralichthys olivaceus)
Source: Biosensors (Basel). 2021 Nov 4;11(11):439. doi: 10.3390/bios11110439 (PMC8615781; doi:10.3390/bios11110439)
Supplement: Supplementary file 1 [file biosensors-11-00439-s001.zip › biosensors-1430684-supplementary.pdf]

biosensors

MDPI

Supplementary Material

Robust Nanozyme-Enzyme Nanosheets-Based Lactate Biosensor for Diagnosing Bacterial Infection in Olive Flounder (*Paralichthys olivaceus*)

Thenmozhi Rajarathinam [1,†], Seonghye Kim [2,†], Dinakaran Thirumalai [1,†], Sujin Lee [2], Minho Kwon [3], Hyun-jong Paik [3], Suhkmann Kim [2] and Seung-Cheol Chang [1,*]

[1] Department of Cogno-Mechatronics Engineering, College of Nanoscience and Nanotechnology, Pusan National University, Busan 46241, Korea; thenmozhi@pusan.ac.kr (T.R.); dinakaran@pusan.ac.kr (D.T.)
[2] Department of Chemistry, Pusan National University, Busan 46241, Korea; seonghyeee@pusan.ac.kr (S.K.); isujin@pusan.ac.kr (S.L.); suhkmann@pusan.ac.kr (S.K.)
[3] Department of Polymer Science and Engineering, Pusan National University, Busan 46241, Korea; mhkwon89@pusan.ac.kr (M.K.); hpaik@pusan.ac.kr (H.-j.P.)
* Correspondence: s.c.chang@pusan.ac.kr
† These authors contributed equally to this work.

S1. PB electrodeposition

Screen-printed carbon electrodes (SPCEs, 4 mm diameter; model C11L) were acquired from Metrohm DropSens (Oviedo, Spain). The surfaces of the SPCEs were cleaned electrochemically in phosphate-buffered solution (PBS; 100 mM, pH 7.0) in 40 cycles between −0.6 and +1.6 V at a sweep rate of 0.1 V s^{-1}. Then, PB nanozyme electrodeposition was carried out by keeping the SPCEs at a fixed potential of +0.4 V for 1 min in 5 mM solutions of $FeCl_3$ and $K_3[Fe(CN)_6]$ with HCl and KCl as supporting electrolytes. Subsequently, the deposited PB nanozyme layer was activated in the supporting electrolyte mixture by polarizing the potential from −0.05 to 0.35 V at a rate of 50 mV s^{-1}. The modified biosensor was named PB/SPCE [S1].

S2. Synthesis of P-rGO

Synthesis of LAHEMA: 2-Hydroxyethyl methacrylate (HEMA; 5.82 mmol), 4-dimethylaminopyridine (DMAP; 0.969 mmol), and lipoic acid (LA; 4.85 mmol) were injected into a 100 mL round-bottomed flask, dissolved in dichloromethane (DCM; 15.0 mL) with a N_2 gas purge, and kept at 0 °C. A solution of N,N'-dicyclohexylcarbodiimide (DCC; 5.82 mmol) in DCM (85.0 mL) was slowly injected dropwise, followed by stirring at 25 °C for 24 h. The final precipitate was collected using a nylon filter. The samples were washed with deionized water, dried with $MgSO_4$, and vacuum-evaporated to remove organic impurities. Finally, the product LAHEMA was obtained.

Synthesis of PSSL: Azobisisobutyronitrile (AIBN; 0.0425 mmol) was injected into a 100 mL round-bottomed flask, which was then backfilled with N_2 gas using a vacuum pump. Sodium 4-styrenesulfonate (SS; 7.64 mmol), LAHEMA (0.850 mmol), and dimethyl sulfoxide (DMSO; 15.0 mL) were injected into the flask and stirred at 90 °C for approximately 3 h. After polymerization, the solution was precipitated using acetone. The mixture was then centrifuged and dried at 25 °C under vacuum overnight to obtain poly(SS-r-LAHEMA), which is abbreviated as PSSL.

In a 500 mL round-bottomed flask, graphene oxide (GO; 40.0 mg) was dispersed in deionized water (400 mL) with mild shaking; then, PSSL (400 mg) was added. Hydrazine monohydrate (4.00 mL) was slowly injected. The resulting mixture was sonicated using a bath-type sonicator (400 W) for 30 min, and then stirred in an oil bath at 70 °C for 6 h. The

solution was sonicated for 30 min, the obtained product was centrifuged at 33,800× *g* for 30 min to remove the free polymers, and the pellet was collected [S2].

Table S1. The concentrations of metabolites acquired by ^{1}H-NMR spectroscopy from the spleen of olive flounder.

Metabolites	Concentration (mM, Mean ± SD)	
	Control	*S. parauberis*
**Acetate**	0.02 ± 0.004	0.023 ± 0.008
**Alanine**	0.035 ± 0.012	0.042 ± 0.009
**Aspartate**	0.019 ± 0.011	0.032 ± 0.008
**Choline**	0.013 ± 0.004	0.007 ± 0.006
**Creatine**	0.026 ± 0.007	0.031 ± 0.009
**Formate**	0.021 ± 0.006	0.02 ± 0.01
**Glutamate**	0.052 ± 0.015	0.067 ± 0.011
**Glutarate**	0.012 ± 0.005	0.09 ± 0.035
**Glycine**	0.013 ± 0.008	0.012 ± 0.008
**Inosine**	0.014 ± 0.006	0.007 ± 0.004
**Isoleucine**	0.008 ± 0.007	0.009 ± 0.004
**Lactate**	0.014 ± 0.006	0.035 ± 0.008
**Leucine**	0.02 ± 0.014	0.02 ± 0.01
**Lysine**	0.018 ± 0.015	0.015 ± 0.012
**Methionine**	0.007 ± 0.005	0.006 ± 0.004
**O-Phosphocholine**	0.004 ± 0.001	0.007 ± 0.002
**Succinate**	0.002 ± 0.0005	0.005 ± 0.003
**Taurine**	0.401 ± 0.076	0.472 ± 0.102
**Threonine**	0.011 ± 0.007	0.01 ± 0.005
**Valine**	0.016 ± 0.012	0.016 ± 0.008
**myo-Inositol**	0.014 ± 0.003	0.013 ± 0.003

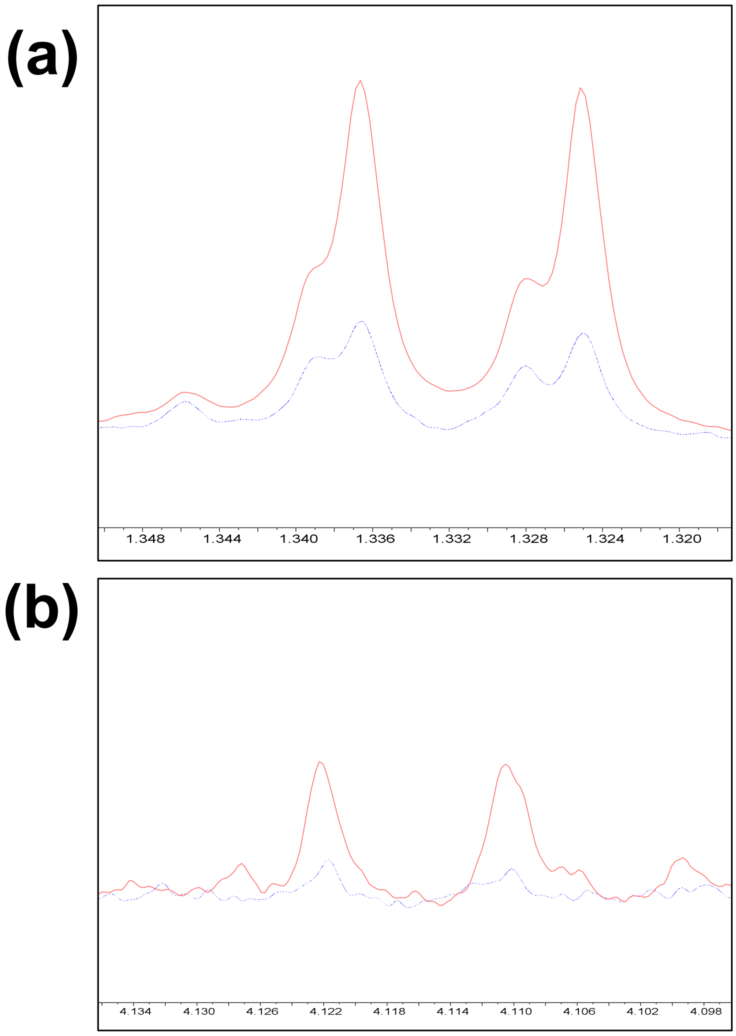

Figure S1. The ^{1}H-NMR spectra of spleen samples from the control (blue line) and *S. parauberis*-infected fish (red line). (**a**) Lactate is represented as a doublet peak at 1.33 ppm (1.326 and 1.338 ppm) and (**b**) a quartet peak at 4.1 ppm in the ^{1}H-NMR spectra.

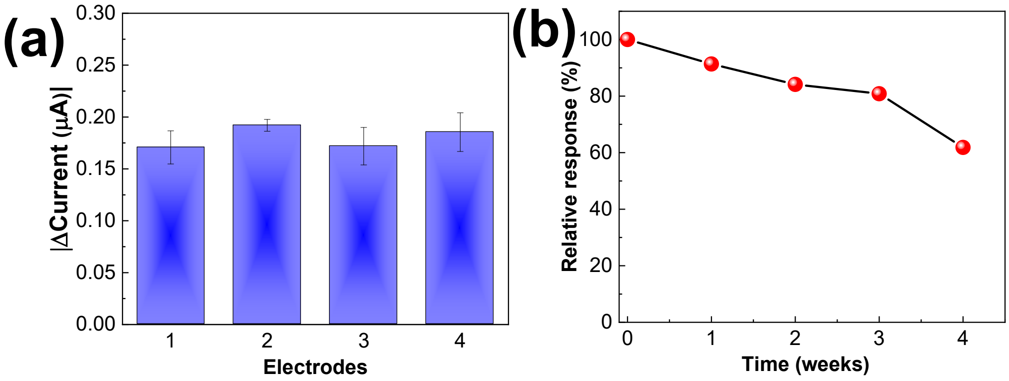

Figure S2. (**a**) Reproducibility of LOXENs/PB/SPCE in 100 μM lactate prepared in 50 mM PBS (pH 7.4). The error bars indicate the mean of four replicate measurements made using four individual biosensors. (**b**) Stability of LOXENs/PB/SPCE after storage at 4 °C over a period of 4 weeks.

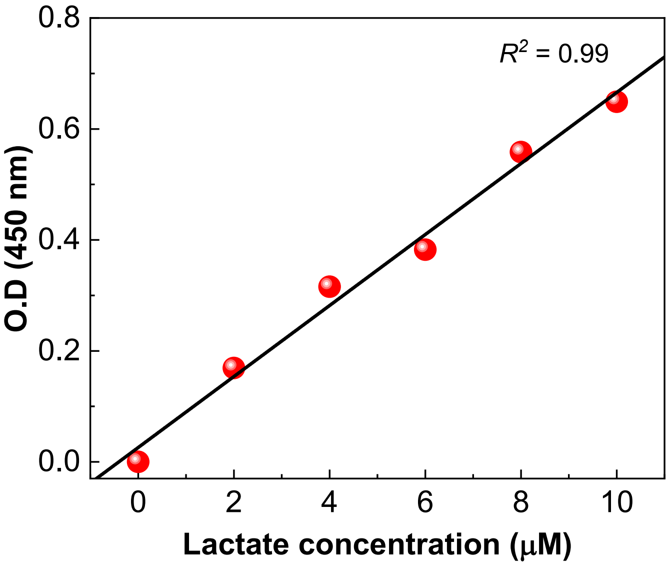

Figure S3. Typical calibration curve of lactate standard obtained using a calorimetric assay kit after background signal subtraction (n = 2 ± SD).

References

[1] Thirumalai, D.; Kim, S.; Kim, S.; Chang, S.-C. Reagentless Amperometric Pyruvate Biosensor Based on a Prussian Blue-and Enzyme Nanoparticle-Modified Screen-Printed Carbon Electrode. *ACS omega* **2020**, 5, 30123–30129, doi:10.1021/acsomega.0c04522.

[2] Rajarathinam, T.; Kwon, M.; Thirumalai, D.; Kim, S.; Lee, S.; Yoon, J.-H.; Paik, H.-j.; Kim, S.; Lee, J.; Ha, H.K. et al. Polymer-dispersed reduced graphene oxide nanosheets and Prussian blue modified biosensor for amperometric detection of sarcosine. *Anal. Chim. Acta* **2021**, 338749, doi:10.1016/j.aca.2021.338749.
